# Towards personalized tumor markers

**DOI:** 10.1038/s41698-017-0021-2

**Published:** 2017-05-25

**Authors:** Vathany Kulasingam, Ioannis Prassas, Eleftherios P. Diamandis

**Affiliations:** 1 Department of Clinical Biochemistry, University Health Network, Toronto, ON Canada; 2 Department of Laboratory Medicine and Pathobiology, University of Toronto, Toronto, ON Canada; 3 Department of Pathology and Laboratory Medicine, Mount Sinai Hospital, Toronto, ON Canada

## Abstract

The cancer biomarker discovery pipeline is progressing slowly. The difficulties of finding novel and effective biomarkers for diagnosis and management of cancer patients are well-known. We speculate that it is unlikely to discover new serological biomarkers characterized by high sensitivity and specificity. This projection is supported by recent findings that cancers are genetically highly heterogeneous. Here, we propose a new way of improving the landscape of cancer biomarker research. There are currently hundreds, if not thousands, of described biomarkers which perform at high specificity (> 90%), but at relatively low sensitivity (< 30%). We call these “rare tumor markers.” Borrowing from the principles of precision medicine, we advocate that among these low sensitivity markers, some may be useful to specific patients. We suggest screening new patients for hundreds to thousands of cancer biomarkers to identify a few that are informative, and then use them clinically. This is similar to what we currently do with genomics to identify personalized therapies. We further suggest that this approach may explain as to why some biomarkers are elevated in only a small group of patients. It is likely that these differences in expression are linked to specific genomic alterations, which could then be found with genomic sequencing.

## Introduction

Tumor markers have been used in oncology for about half a century. Their discovery in the mid-1960s and 1970s sparked enthusiasm that such molecules could be used to combat cancer through screening, early diagnosis, monitoring of therapy, prognosis, and prediction of therapeutic response. The suggestion that screening for early disease detection could change the course of cancer, thus more people would be cured by early interventions, proved to be partially true. While for some cancers screening is clearly beneficial (such as colon and cervical cancer),^1^ for other cancers, screening is not effective. Some major cancers (such as breast and pancreatic) proliferate quickly and when the cancers are detected by screening, they have already spread.^2^ On the other hand, slow growing cancers are not usually lethal and their detection may lead to over-treatment, which has its own side-effects.^1, 3^ These caveats underline the need for finding tumor markers with outstanding analytical and clinical performance (high sensitivity, specificity, predictive value).

## Reasons for biomarker failures

In 1998, the National Institutes of Health Biomarkers Definitions Working Group defined a biomarker as “a characteristic that is objectively measured and evaluated as an indicator of normal biological processes, pathogenic processes, or pharmacologic responses to a therapeutic intervention. In this manuscript, we will limit our discussion to serological biomarkers, which are usually protein molecules circulating in blood at abnormal amounts due to the presence of a tumor. We will not focus on genomic changes which could be used for cancer diagnosis, prognosis or prediction of therapy, although some comments apply to these biomarkers as well.

The current clinically used serological tumor markers (about a handful) were discovered at least 30 years ago. No major serological tumor markers have been introduced to the clinic since then (although a few genomic markers were Food and Drug Administration (FDA)-approved for predicting therapeutic response). We and others previously identified three reasons of newly discovered biomarker failures^4–6^: (a) fraudulent publications (very rare). (b) discovery of markers with weak performance characteristics such as low sensitivity, specificity or predictive value, precluding their clinical utility. (c) false discovery, i.e., reports on tumor markers, which initially promise to revolutionize cancer management but which subsequently fail rigorous validation. Some reasons, and examples of false discovery, have been described in our previous communications.^4–6^ False discovery is closely related with the issue of irreproducibility in science, a highly debated contemporary topic.^7, 8^

The Early Detection Research Network (EDRN) (https://edrn.nci.nih.gov/) was mandated by the National Cancer Institute of USA to discover, validate and promote biomarkers for early diagnosis. During the last 15 years, EDRN spent significant funds (> $100 million) to support biomarker discovery and validation laboratories, and to help transition biomarkers from the lab to the clinic. The outcomes have been rather modest. Most successfully validated biomarkers by EDRN originated from discoveries in industry. Extensive validations by EDRN investigators and others of hundreds of cancer biomarkers revealed that in general, the newer putative markers are not as good as the traditional ones for any of the intended clinical applications (including screening and early diagnosis).^9–11^

## Undiscovered, highly sensitive biomarkers are unlikely to exist

Why is it proving extremely difficult to find new cancer biomarkers with adequate sensitivity and specificity to be used in the clinic? The history of cancer biomarkers can provide some important lessons. First, we now know that each site-specific cancer has histological sub-types with different origins and mutational spectra, such that the subtypes can be considered different diseases. Second, the latest genomic advances in oncology are suggesting that tumors are highly heterogeneous, and no two tumors (with some exceptions) have the same mutational spectrum.^12^ Even within the same tumor, molecular heterogeneity is enormous and differences can be seen in primary vs. metastatic sites or as tumors evolve over time.^13, 14^ These new findings support the view that it is highly unlikely to identify a single marker which will be elevated in nearly all patients with a specific malignancy. How then are some currently used tumor markers elevated in most patients, especially at the advanced stages? Among the reasons are the following:

1. Some markers are tissue-specific (such as prostate-specific antigen (PSA)) and their elevation in serum is due to leakage of the highly abundant PSA molecules in prostate cells (benign or malignant) into the adjacent blood vessels.^15^ Circulating tumor DNA is a similar example of an elevation of a biomarker in nearly all patients with cancer, especially at late stages.^16^ This test is highly sensitive and specific, since the DNA leaks from dying tumor cells. In the case of PSA, this molecule is not known to be involved with the initiation or progression of prostate cancer.^17^ Normal and cancer cells make about the same amount of PSA. These facts also explain why PSA is elevated in non-malignant diseases, such as prostatitis and benign prostatic hyperplasia.
2. Some other clinically used tumor markers (e.g.: carcinoembryonic antigen; alpha-fetoprotein) are elevated in the majority of patients with cancer because they represent onco-fetal antigens: molecules which were expressed at high levels in fetal life and then re-expressed again in malignancy due to the immature nature (de-differentiation) of cancer cells.
3. A third class of biomarkers that are elevated in many patients include the carbohydrate antigens, highly glycated molecules involved in the ubiquitous processes of cell adhesion and barrier function.

Since all clinically used cancer biomarkers were discovered at least 30 years ago, by using minimally sophisticated techniques (compared to contemporary methods), it is reasonable to speculate that similarly performing molecules are unlikely to await future discovery. This view is further reinforced by the fact that new and highly powerful databases exist for assisting with biomarker discovery but success is still modest. Such databases include The Protein Atlas,^18^ The Cancer Genome Atlas^19^ and the International Cancer Genome Consortium.^20^

## Rare tumor markers

The literature is full of reports on new cancer biomarkers with weak clinical characteristics, thus precluding their widespread use in clinical practice. For example, at a certain cutoff, a cancer biomarker could have a sensitivity of 5, 10, 20 or 30%, at a reasonably high specificity (i.e., ≥ 90%). Such biomarkers are currently not considered clinically useful, or worth commercializing, due to their low sensitivity.

We previously reported that some biomarkers, such as human kallikreins 6 and 10 (KLK6 and KLK10) are consistently elevated in about 2–5% of pancreatic cancer sera, at 100% specificity,^21^ leading us to query if such biomarkers have any role to play in clinical practice. We advocated that it may be possible to develop a repository of “rare tumor markers” (i.e., those with low sensitivity but high specificity) which, individually, are not highly useful. We then suggested that these markers may be useful in selected patients (see below).

Our suggestion is analogous to the initiative developed by the National Cancer Institute a few years ago, known as “exceptional responders in clinical trials”.^22^ Exceptional responders are defined as individuals who have a favorable response lasting at least 6 months in a clinical trial for a drug that was not approved for that cancer because very few patients responded overall. The “rare responders” database hopes to help understand why certain patients responded to obtain insights on the biological behavior of these tumors. Already, important lessons have been learnt through studying these rare responders.^23^ The basis of this so-called “precision medicine” is similar, because it aims to identify groups of patients who may have better therapeutic responses to specific drugs than other patients.^24^

## Repository of rare tumor makers

We invite cancer biomarker researchers to submit their “rare tumor markers” as broadly defined above (< 30% sensitivity at > 90% specificity) for inclusion into an open access database. To facilitate uniform submissions and inclusion of critical information, we created a “submission form” (supplementary information). The provided information should be enough for independent data reproduction. We envision that such a database will have a few broad applications:

1. Through this database, researchers could identify candidate tumor markers, which are informative for one or a few patients; then use the biomarker to guide patient management.
2. The data would facilitate further investigations (e.g., whole-genome sequencing),^25^ to delineate as to why a particular tumor produces the aforementioned biomarker. This information may enrich our knowledge about tumor biology and may pinpoint new therapeutic targets.

## Centralized testing laboratory

In the near future, we envision the creation of centralized laboratories to develop and validate highly robust assays for rare tumor markers. Such laboratories could seek clinical laboratory improvement amendments certification and FDA approval of their tests, to ensure high quality results.

**Fig. 1:**
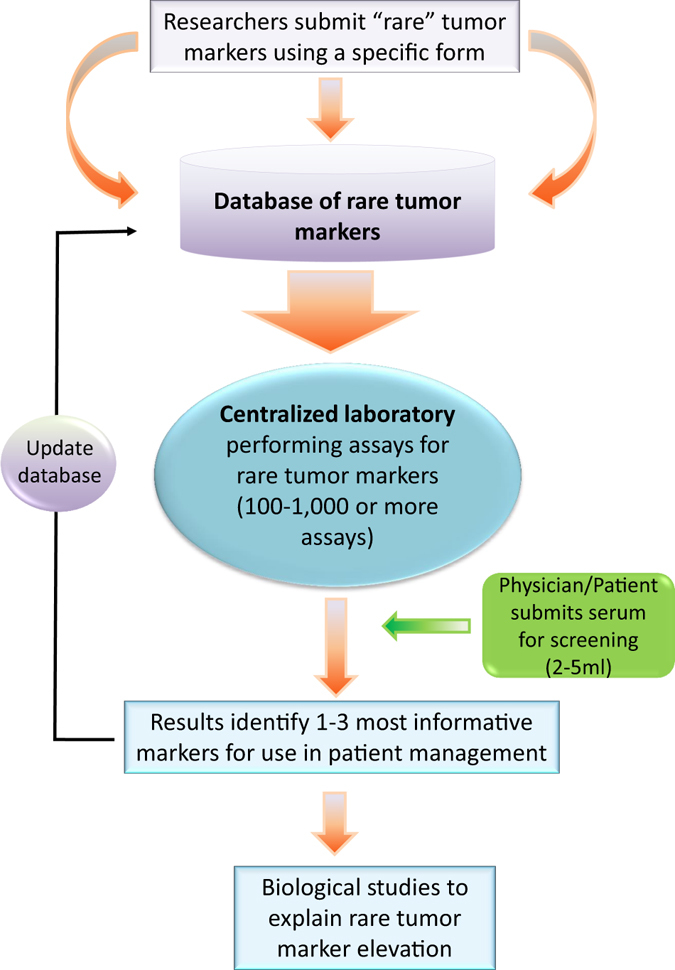
Personalized tumor marker database and clinical use. See text for more details

Patients/physicians could submit a serum sample from newly diagnosed cancer patients to be used for screening 100–1000 candidates, with a goal to identify 1–5 biomarkers that are most informative for these patients and thus use them for management (Fig. 1). Preferably, submitted samples are collected before initiation of any treatment and after 4–6 weeks post treatment, to identify molecules that are altered by treatment. Although initially, this facility may have a limited assay menu, it is conceivable that over 1–3 years, enzyme-linked immunosorbent assay (ELISA) assays for at least 1000 or more markers could be developed. While tumor marker types include proteins, DNA, transcripts, metabolites, cells, etc. our discussion here focuses only on circulating biomarkers in serum. Other types of tumor markers could also be similarly utilized. Due to tumor heterogeneity during evolution and metastasis, the characteristics of these rare tumor markers may need to be reassessed with additional screens.

Facilities or technologies which can provide quantitative information on thousands of proteins are becoming available now. For example, some companies already offer quantitative assays for thousands of proteins in micro-ELISA array formats using small volumes (< 2 ml) (e.g., see www.raybiotech.com). Also, mass spectrometric selected reaction monitoring assays for entire human and other proteomes have been published.^26, 27^ The latter technology still suffers from sensitivity issues but these difficulties are expected to be solved or improved soon.

## Highly sensitive assays

One possible limitation of our suggestion is the amount of serum necessary to screen 500 or 1000 biomarkers. One solution includes multi-parametric assays, such as the Luminex platform^28^ (see also www.abcam.com). Recently, other options have emerged. A few companies have recently developed ultrasensitive ELISA assays for many analytes.^29^ For example, MesoScale’s fifth generation complexed PSA assay has a sensitivity of 6 fg/ml, which allows quantification of serum PSA in all women.^30^ While such extreme sensitivity is usually not necessary to quantify a single biomarker, the technology allows for significant sample dilution before the final testing. For example, serum from a normal male can be diluted 1000–10,000 times and is still easily measurable for PSA with such assays. Since most of the known and newly reported cancer biomarkers exist in the circulation at levels of 1 pg/ml or higher, these technologies would allow a 100-fold dilution of the sample before analysis. Consequently, 2–5 ml of serum would be enough to screen 1000 or more analytes.

## Outlook

We believe that this suggestion could open-up a new era in cancer biomarkers (Fig. 1). Biomarkers that are currently deemed useless could find utility in specific patients, the same way as “personalized therapies” do. The systematic cataloging and assay of many tumor markers for every cancer site will eventually obviate the problem of finding (if they exist) biomarkers with very high sensitivity and specificity for all patients.

Our general suggestion has similarities to other clinical settings. For example, in the area of transplantation, donors and recipients are first screened for human leukocyte antigen histocompatibility in a centralized laboratory, to avoid graft rejections. Last, but not least, we caution that our suggestion has not as yet been tested in practice and its effectiveness needs to be verified with experimental data. On some occasions, similar strategies identified candidate biomarkers for Alzheimer’s disease,^31^ traumatic brain injury,^32^ and cancer.^33–36^

## Electronic supplementary material

Supplemental Material

## Electronic supplementary material

Supplementary Information accompanies the paper on the *npj Precision Oncology* website (doi:10.1038/s41698-017-0021-2).
