# Supplementary material for: Towards personalized tumor markers
Source: NPJ Precis Oncol. 2017 May 25;1:17. doi: 10.1038/s41698-017-0021-2 (PMC5871887; doi:10.1038/s41698-017-0021-2)
Supplement: Supplementary file 1 — Supplemental Material [file 41698_2017_21_MOESM1_ESM.pdf]

Supplementary Table 1: Information to be included when submitting a 'rare' tumour marker [1,2]

------------------------------------------------------------------------------------------------------

1) Official name and aliases of the 'rare' tumour marker

2) Sensitivity and specificity for a cancer site (many sites can be mentioned) and cutoffs used.

3) Mention distribution of marker according to clinical stage, if applicable.

4) Cite literature reference, if data are published.

5) Describe patients and clinical samples used, such as inclusion criteria for cancer and non-cancer groups (controls). Sample type, length of storage, freeze-thaw cycles, analyte stability, effect of hemolysis/lipemia, patient age and gender.

6) Describe assay used (commercial or other source), antibodies, their specificity, any known cross-reactivity, analytical precision and sensitivity, hook effect. Cite literature or commercial kit insert. Describe other assay types (eg. SRM mass spectrometery) in sufficient detail to allow reproduction.

7) Any other information that could allow reproduction of data

------------------------------------------------------------------------------------------------------

[1] The broad definition of a 'rare' tumour marker is <30% sensitivity and >90% specificity at a specified cut-off value.

[2] Consult other guidelines such as STARD (35) and many others, as summarized in Ref. 36
